# Which educational messengers do medical students prefer for receiving healthinformation? Development and psychometrics of using health messengers questionnaire

**DOI:** 10.1186/s12889-023-17400-1

**Published:** 2024-01-10

**Authors:** Zahra Karimian, Mehrvash Moradi, Nahid Zarifsanaiey, Sara Kashefian-Naeeini

**Affiliations:** 1 Ph.D. of Higher Education Administration and Msc in Medical Education, Associate Professor, Department of E-Learning in Medical Sciences, Virtual School and Center of Excellence in E-Learning, Shiraz University of Medical Sciences, Shiraz, Iran; 2 MSc of e-Learning in Medical Sciences, Virtual School and Center of Excellence in E-Learning, Shiraz University of Medical Sciences, Shiraz, Iran; 3 Ph.D of Distance Education, Professor, Department of E-Learning in Medical Sciences, Virtual School and Center of Excellence in E-Learning, Shiraz University of Medical Sciences, Shiraz, Iran; 4 Ph.D. of TESL, Assistant professor, Department of English Language, School of Paramedical Sciences, Shiraz University of Medical Sciences, Shiraz, Iran; 5 Philosophy of Life and Healthy Lifestyle Research Center, Shiraz University of Medical Sciences, Shiraz, Iran

**Keywords:** Messenger, Student, Health information, Preferences, Social media, Medical sciences

## Abstract

**Introduction:**

Individuals vary in their selection of health messengers. This research aimed to construct an instrument to measure the preferences of medical students in selecting health messengers and in the next step to validate the aforementioned questionnaire.

**Method:**

This research is a descriptive survey with an approach to construct a questionnaire. The statistical population included all students studying at Shiraz University of Medical Sciences in March to June 2022 in the academic year 2021-2022. 500 participants were involved in the study. To determine the types of health messengers and review the texts, a group of 15 primary items consisting of the 6 components of academic sources (2-items), formal news sources (2-items), mass media (3-items), internet search (2-items), social networks and messenger applications (4-items), and informal conversation (2-items) were compiled. A 4-point scale was developed the content validity of which was confirmed using CVI and CVR method and the reliability index was calculated to be 0.818. Factor analysis was also used to determine the construct validity and factor loading of each item.

**Results:**

The research covers university students in different medical fields. Using factor analysis, together with KMO = 0.810 and Bartlett's sphericity index P < 0.0001, saturation and the suitability of the test were confirmed. Students' preferences based on factor load were social media (28.92%), official and unofficial health sources(10.76%), academic sources (9.08%), internet search (8.18%), and mass media (7.13%), respectively. Among social media, Telegram (0.85) had the highest factor load followed by Instagram (0.79), and WhatsApp (0.71).

**Conclusion:**

Medical students are always on the move and naturally prioritize mobile-based methods. They prefer messengers that are free from time and space restrictions. The widespread availability of mobile devices and the ability to search for and access information make it easier to test health information. Therefore, in health policy, attention should be paid to the virtual capabilities, especially mobile-based approaches.

## Introduction

The media provides people with a variety of information in the form of messages. Media outlets think, justify, change, replace, and make decisions, and by repeating and inculcating, they institutionalize new beliefs and attitudes among people [1]. The media performs this task by designing messages for people. Among the types of messages that are distributed in the media, those messages that directly or indirectly discuss people’s personal and social health, including living a healthy life and taking prevention and treatment measures, are regarded as health messages [2].

During the last few decades, the media have been used to exert influences on various health behaviors in people, and they have often functioned with the aim of reducing smoking habits or preventing heart diseases. Moreover, issues such as prevention of alcohol and drug abuse, screening and cancer prevention, sexual behaviors, children's health and many other health-related issues have been impacted by the media. These messages have been transmitted through a variety of media including television, radio, outdoor media such as billboards and posters, and print media, such as magazines and newspapers, and have gained popularity with the emergence of new technologies such as the internet, mobile phones, or online searches on websites [3, 4]. Various types of health messages in health communication can be divided in the forms of instructional, awareness, social or persuasive messages. Each of them has an impact on the development of health awareness in a society.

Maintaining students’ health and quality of life is of paramount importance since this group plays an important role in the future management of countries [5]. Students’ health habits and behaviors will have a great impact on their quality of life in the future [6]. However, as students are often considered to be in a relatively healthy state of life, less attention is paid to health promotion programs and this dilemma is common all over the world [7]. Due to their youth, students often assume that diseases occur in old age and think less about their health [8]. Meanwhile, research shows that unhygienic behaviors have become prevalent among this important group of the society. Some studies have shown that students accept less responsibility for their health [9], often skip breakfast from their daily meals [10], have unhealthy foods [11], or have a sedentary lifestyle [12, 13]; they are also more prone to unhealthy lifestyles, the tendency to smoke, an unhealthy diet and increased stress [13].

The previous studies in Iranian universities indicate that students are not making good choices in healthy lifestyle habits such as nutrition, physical activity, and other health indicators [14]. Even in medical universities where students are studying health-related professions, they may not have a good score in terms of a healthy lifestyle [15]. It is crystal clear that this problem is not limited to a specific region of the world, and the evidence suggests that it is a significant global issue that may be influenced by various variables. In a study conducted on medical university students in Saudi Arabia, the results showed that the average health-promoting lifestyle score of the students was not satisfactory [16]. Two other studies conducted on university students in the United States [17], and the United Kingdom [18] also showed similar results with their health indicators being lower than expected. All these evidences clarify that paying attention to promoting and maintaining a healthy lifestyle for students is very important and worth considering.

The most important strategies in improving the quality of life is education, awareness and people’s knowledge. Today, in many countries in the world, global health coverage is one of the main projects and indicators of sustainable development until 2030, with an emphasis on education and improving people's knowledge and awareness [19]. Meanwhile, the media is the main platform for the development of health communication and its use affects the health behaviors [20, 21]. Social media can partially intervene in individual behaviors and cover various disease prevention behaviors such as physical fitness and exercise, anti-smoking behaviors, and the prevention of AIDS [22].

The relationship between social media and health information had been investigated and it was found that, surprisingly, information is presented through new media, and in particular, hilarious information could grab users’ attention, and educational information was disseminated through users [23]. The health literacy of some residents of Beijing, China was also studied and it came to light that health behaviors were positively associated with frequent use of traditional media such as newspapers and television [24]. People who use more media related to health information have positive attitudes towards health [25]. In addition, people may receive health information from different ways such as books, articles, family or social networks [26–28]. In one categorization, health information sources can be divided into two groups of official sources (physicians and health service providers) and unofficial sources (family members and mass media) [1]. However, the emergence of the internet makes people have easier and faster access to the health information, which in turn helps them manage their health [29]. As a new media, the internet can spread health-related issues and information in various ways [30]. It can force different target groups or individuals to accept the knowledge presented in the field of health and make the promotion of public health possible. A large number of people use the m-Health platform and social networks to receive health-related information, health self-efficacy, and social support [31].

Mobile applications are practical programs designed for users so they can be installed on electronic devices such as smart-phones and tablets [32]. A messaging application is a group of applied programs or applications the subscription to which help individuals communicate with others and discuss and exchange opinions, share the images and videos they like, and get the opportunity to comment on other people's interests and content. They can also share their content according to their taste and opinion [33]. Among these apps, one can mention Whatsapp, Telegram and Instagram. This group of messengers are platforms for transferring and strengthening health education [34] and since they are of the same generation as students and young people, they are among the main choices of this group in exchanging information, including health information and healthy behaviors. In general, students receive messages and health information in different ways, and in the process of transmitting the message, the source of the message is considered one of the most important elements of this process. Selecting the right source can increase the effect of the message [35]. Beyond doubt, media is the most important tool for conveying messages, and having a correct understanding of media is crucial for developing health in society. Different individuals and groups have different preferences based on age, education, gender, and living conditions. One of the most important contents that people search for is health-related information. Medical students search for health information to improve their knowledge and to transmit it to patients, people, and families, making the credibility of these sources doubly important. Therefore, there is a need for a tool to evaluate medical students' preferences in selecting health media.

The current research has been carried out with the following objectives:

- Designing and psychometric analysis of the UHMQ questionnaire components
- Determining students' preferences in choosing health messengers

## Methods

### Research design

The study is a descriptive survey with an approach to construct a research instrument. The research was carried out at Shiraz University of Medical Sciences (SUMS). The statistical population of this research includes all students studying at SUMS in 2021–2022.

### Participants

The participants consisted of all undergraduate and postgraduate students at SUMS in the academic year 2021–2022. The inclusion criteria were being enrolled as a student at SUMS, voluntarily participating in the research, and having access to a mobile device. The exclusion criterion was samples that had not responded to more than 20% of the questions.

### Sampling

The study population of this research encompasses all medical students in SUMS in 2021–2022, which amounted to approximately 5000 individuals. Cochran's formula (29) was used, considering the following values: N = 5000, study confidence level = 95%, estimated error rate = 0.05, and z value = 1.96. The approximate values of p and q were both 0.5. Based on these parameters, the sample size was calculated using the formula. Confidence interval was 95%.

$$n=\frac{\frac{{{\text{Z}}}^{2}pq}{{{\text{d}}}^{2}}}{1+\frac{1}{N}\left(\frac{{{\text{Z}}}^{2}pq}{{{\text{d}}}^{2}}\right)-1}$$

The estimated sample size was around 357 individuals. However, since many studies have reported low response rates for electronic questionnaires, emails were sent to 550 students, and ultimately, 500 complete questionnaires were received (a response rate of approximately 91%.). The sampling method employed was random sampling, achieved by drawing from the list of students’ email addresses.

### Instrument/tool

The research tool used was a researcher-developed questionnaire consisting of 15 items categorized into 6 components. The construction of the questionnaire was conducted in two phases: qualitative and quantitative.

In the qualitative phase, to determine the types of health messengers, first we used studies that were conducted in the literatures [1, 3, 36]. Afterward, using two focus groups consisting of 5 faculty members with specialties in the fields of Medical Education, E-Learning, Educational Technology and Health Management, an initial questionnaire which comprised 15 items was constructed. Based on the initial classification, a questionnaire including 6 components: academic sources (2 items), formal news sources (2 items), mass media (3 items), internet search (2 items), Social Networks and Messenger Applications (4 items), and Informal Conversation (2 items), were prepared and the scale of the questionnaire in the range of always = 4, often = 3, sometimes = 2 and rarely = 1 was applied (the scoring range for the questionnaires was from 1 to 4, with a cutoff point of 2.5).

In the quantitative phase, the validation of the instrument was conducted. Firstly, based on expert opinions, the face and content validities of the instrument were examined. Then, the construct validity of the instrument was assessed by distributing the questionnaires and collecting data from 500 participants. Exploratory factor analysis was performed to examine the structural validity of the instrument. Finally, the reliability of the instrument was measured using internal consistency of the items.

### Validity

#### Face validity

The initial questionnaire was given to 10 educational experts and the face validity of the questionnaires was reviewed. According to the experts’ evaluation, all the questions were appropriate and no changes were suggested in the questions.

#### Content validity

Content validity was determined using the opinions of 10 experts in the field of E-learning and medical education and through the Content Validity Ratio (CVR) and Content Validity Index (CVI). CVR is a content validity measurement approach which was proposed by Lawshe. The experts were asked to score each of the questions based on a three-part Likert scale of absolutely necessary = 3, relatively necessary = 2 and not necessary = 1. Based on the Lawche model, it is expected that by surveying 10 experts, we will have at least 62% agreement [37]. CVI was also used to measure the validity of the questionnaire. This CVI was provided by Waltz & Bausell. To calculate the CVI, the experts were asked to rate each item in the three areas of relevance, clarity, and simplicity in the range of 1 to 4. To calculate the index, we divide the number of people who chose option 3 and 4 by the total number of experts. If the result is greater than 0.79, it is acceptable, although the score between 0.7 and 0.79 can be re-examined and refined [38].

#### Construct validity

Considering that one of the objectives of the current research is the psychometrics of the questionnaire, and this questionnaire has been prepared for the first time, the exploratory factor analysis method was used for construct validity, the detailed results of which are presented in the results section.

#### Reliability

To determine the reliability, internal consistency was used. In addition to assessing the overall reliability, the reliability of the questionnaire dimensions (components) was also determined. Furthermore, the reliability of each item was examined if it was deleted. This method helps to investigate the impact of each item on the overall reliability. If removing an item leads to an increase in reliability, it indicates that the item has a detrimental effect on the overall reliability and may require reconsideration.

### Data collection

The questionnaire was designed in an electronic format. In the introduction to the questionnaire, the research objectives were outlined to the students, and they were asked to indicate their preferences regarding the use of various health messaging media by selecting their choices. The questionnaire link was sent to the students via email. Since the questionnaire was electronic, there was a potential for participants to respond multiple times or leave incomplete responses. Therefore, during the data extraction process from the system, only the most recent questionnaire was retained for those participants who submitted multiple responses from the same IP address at the same time. However, it is important to note that in this study, we only had three incomplete questionnaires without any identifiable information, which were excluded from the analysis.

The questionnaires were distributed in the second half of the academic year, and the data collection period ranged from March to June 2022. The questionnaires were collected and analyzed anonymously, viz. no personally identifiable information was associated with the responses during the data collection and analysis procedures.

### Data analysis

The data were analyzed using SPSS 24 software. Exploratory factor analysis method was used for the psychometric measurement of the instrument (Construct validity).

## Results

Based on the findings of the research, a total of 500 questionnaires were collected. 200 (40%) male students and 300 (60%) female students participated in the study. The age range was between 18 to 65 years old and the average age was about 27.25 + 7.85.

**Fig. 1:**
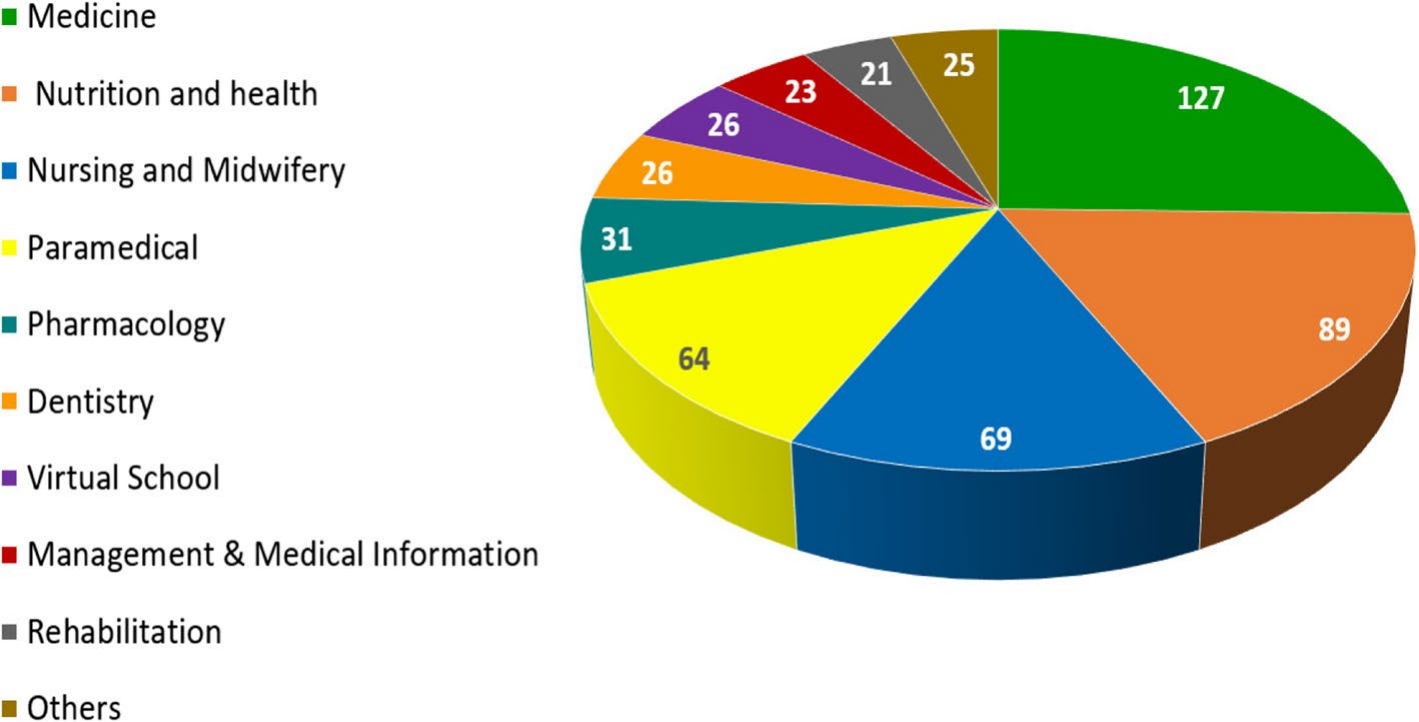
Distribution of the frequency (Number) of participants by faculty

Most of the participants were undergraduate students (N = 181) which amounted to 36.4% (See Table 1). The distribution of participants of different schools are illustrated in Fig. 1.

**Table1:** Descriptive information on demographic characteristics of participants

Characteristics		Frequency	
		N	%
Gender	• Male	200	40
	• Female	300	60
	• Total	500	100
Age	• 18 < Year < 25	276	55.8
	• 26 < Year < 35	147	29.7
	• 36 < Year	72	14.5
	• Total	495	100.0
Field of Study	• Clinical(Medicine and Dentistry)	125	25.0
	• Basic medical scienc (Biochemistry, Immunology, Physiology, Anatomy…)	85	17.0
	• Para Medical (Nursing, Midwifery, Health care, Physiotherapy,…)	232	46.5
	• None Medical Sciences(Computer, English language, Education,…)	58	11.6
	• Total	500	100.0
Grade	• BSc	181	36.4
	• Proffesional Doctotare	138	27.8
	• MSc	102	20.5
	• Ph.D/ Clinical Residents	76	15.3
	• Total	497	100.0
Marital status	• Single	366	73.3
	• Married	134	26.7
	• Total	500	100.0
Residential status	• With parents	219	43.8
	• Independent	137	27.4
	• Dormitory	144	28.8
	• Total	500	100.0

### Instrument psychometric analysis

#### Face and content validity

Before distributing the questionnaire, a 15-item scale was constructed based on theoretical foundations, previous research and consultation with five experts in the fields of Medical Education, E-learning, Educational Technology and Health Management. It was categorized into six primary components based on the commonality of concepts. In reviewing the face validity, all the items were confirmed and no changes were recommended in the questionnaire. Then, to determine content validity, the questionnaire was given to ten faculty members in the fields of Health Education and Educational Technology (Table 2).

**Table 2:** Primary UHMQ items based on articles and opinions of the focal group

Components	Items
Academic Resource	1. Scientific books and articles
	2. Scientific conferences, webinars and meetings
Formal News and Information	3. Health messages and news from the Ministry of Health (SMS)
	4. The website of the SUMS or the Ministry of Health
Mass Media	5. Radio
	6. Television
	7. Public Magazines and newspapers
Internet Search	8. Search health related websites
	9. Search for information on Google
Social Networks and Messenger Applications	10. Health-related groups on WhatsApp
	11. Health-related groups on Instagram
	12. Health-related groups on Telegram
	13. Health-related groups on local messengers
Informal Conversation	14. Conversation and exchanging news with the family
	15. Conversation and exchanging news with colleagues and classmates

#### Reliability of the questionnaire

To obtain a measure of internal consistency of the research instrument, Cronbach's alpha was run after collecting the data pertinent to the 500 participants. The alpha coefficient was 0.818 suggesting that the items had a high internal consistency. The results are presented in Table 3.

**Table 3:** Psychometric properties of content validity and reliability of the UHMQ questionnaire

Components	Items	Content Validity				Reliability	
		CVR	CVI			Cronbach's Alpha	
		Essential	Simple	Clear	Relevant	Factors	If Item Deleted
Academic Resource	Q1	1.00	1.00	1.00	1.00	0.675	0.818
	Q2	0.80	0.90	1.00	0.80		0.808
Formal News and Information	Q3	0.60	0.90	1.00	1.00	0.733	0.804
	Q4	1.00	1.00	1.00	1.00		0.798
Mass media	Q5	1.00	1.00	1.00	1.00	0.633	0.816
	Q6	1.00	1.00	1.00	1.00		0.819
	Q7	1.00	1.00	1.00	1.00		0.808
Internet search	Q8	1.00	1.00	1.00	1.00	0.645	0.808
	Q9	1.00	1.00	1.00	1.00		0.815
Social Networks and Messenger Applications	Q10	1.00	1.00	1.00	1.00	0.761	0.793
	Q11	1.00	1.00	1.00	1.00		0.802
	Q12	1.00	1.00	1.00	1.00		0.805
	Q13	0.40	0.80	0.80	0.80		0.807
Informal Conversation	Q14	1.00	1.00	1.00	1.00	0.643	0.803
	Q15	1.00	1.00	1.00	1.00		0.808
Total	CVR _Total_ = 0.920		0.973	0.987	0.973	R _Total_ = 0.818	
			CVI _Total_ = 0.978				

#### Construct validity

Inasmuch as health messengers questionnaire was designed as a researcher-made one, exploratory factor analysis was used to determine the construct validity. In factor analysis, in addition to construct validity, the importance and factor load of each of the messengers was also determined. As was previously mentioned, based on previous research and discussion in the focus group, 15 items and 6 factors were extracted. The table of items and components is given in Table 2.

### Criteria for implementing exploratory factor analysis

#### Variable appropriateness criteria prior to the implementation of exploratory factor analysis

To determine the validity of the obtained results, two important criteria of the test must be examined before implementing the factor analysis:

- 1. The meaningfulness of “Bartlett’s Sphericity Test “which confirms the relative correlation between the variables for the implementation of the test.
- 2. Control of the KMO coefficient, which is an indicator for measuring the adequacy of the number of samples to perform the factor analysis test. In KMO and Bartlett's Test, factor analysis can be performed with confidence when KMO is greater than 0.6 [39]. Amounts above 0.9 are marvelous, between 0.80 to 0.89 are meritorious, between 0.70 to 0.79 are average and between 0.60 to 0.69 are mediocre [40–42].

The results of Bartlett's Sphericity Test and KMO of the present research are shown in Table 4. They indicate that the sample size is suitable and adequate for using the test (KMO = 0.810).

**Table 4:** KMO Index value and Bartlett's Test for Component Analysis

Scale	Results of Bartlett’s Test	KMO		
	Sig		χ 2	df
Health Messengers Components	< 0.0001	0.810	1992	105

### Appropriateness of variables criteria after implementing the factor analysis

#### The criterion of the degree of communalities of items

After confirming the appropriateness of the factor analysis for research and its implementation, it is necessary to review the appropriate variables which are to be kept in the research. To this end, criteria such as the degree of correlation or communalities are used to determine the appropriateness of the variables.

The minimum acceptable criterion for keeping a variable in research is above 0.5 [43]. However, some articles consider amounts above 0.3 to 0.4 as acceptable [36]. In the current research, the appropriateness of the variables to remain in the research was considered to be more than 0.5. The results showed that all items except item 13 had a factor loading of over 0.5. (Table 5).

**Table 5:** The communalities of Items in the questionnaire

Item Number	Extracted	Item Number	Extracted	Item Number	Extracted
Q1	0.674	Q6	0.630	Q11	0.678
Q2	0.729	Q7	0.552	Q12	0.760
Q3	0.686	Q8	0.661	Q13	0.404
Q4	0.643	Q9	0.687	Q14	0.606
Q5	0.659	Q10	0.663	Q15	0.577

#### Criteria for determining the number of factors and factor load

The most important aim of factor analysis is to reduce variables into main factors and classify variables into appropriate and common categories. Based on the "Kaiser" criterion, 5 factors were extracted, while based on the previous research and opinions of the focus group, 6 factors were estimated. Based on the "Kaiser" criterion, only components the squared factor loads or ‘Eigenvalue’ of which are greater than one are acceptable. The number of factors and related factor loads are shown in Table 6.

**Table 6:** Total variance explained

Components	Initial Eigenvalues			Extraction Sums of Square Loadings			Rotation Sums of Squared Loadings		
	% of Variance	Cumulative %	Total	% of Variance	Cumulative %	Total	% of Variance	Cumulative %	Total
**1**	28.92	28.92	4.34	28.92	28.92	4.34	15.49	15.49	2.32
**2**	39.68	10.76	1.61	39.68	10.76	1.61	30.34	14.85	2.23
**3**	48.76	9.08	1.36	48.76	9.08	1.36	42.22	11.88	1.78
**4**	56.94	8.18	1.23	56.94	8.18	1.23	53.21	10.99	1.65
**5**	64.07	7.13	1.07	64.07	7.13	1.07	64.07	10.86	1.63
**6**	69.37	5.30	0.79						

Based on the obtained results, four extracted factors explained a total of 64.07% of the variance of health messengers, that is, based on the analysis of the opinions of the participants of this questionnaire in point 1.07, the five main components explained or fitted more than 64% of the concept of health messengers. The amount of this variance and the alignment of the results of the exploratory factor analysis with the theory indicate the appropriate validity of the tool. According to the results obtained in Table 6, the first component (Social Networks and Messenger Applications) had the highest factor loading of 28.92%, followed by Formal and Informal Messengers (10.76%), Academic Resources (9.08%), Searching the Internet (8.18%), and Mass Media (7.13%).

**Fig. 2:**
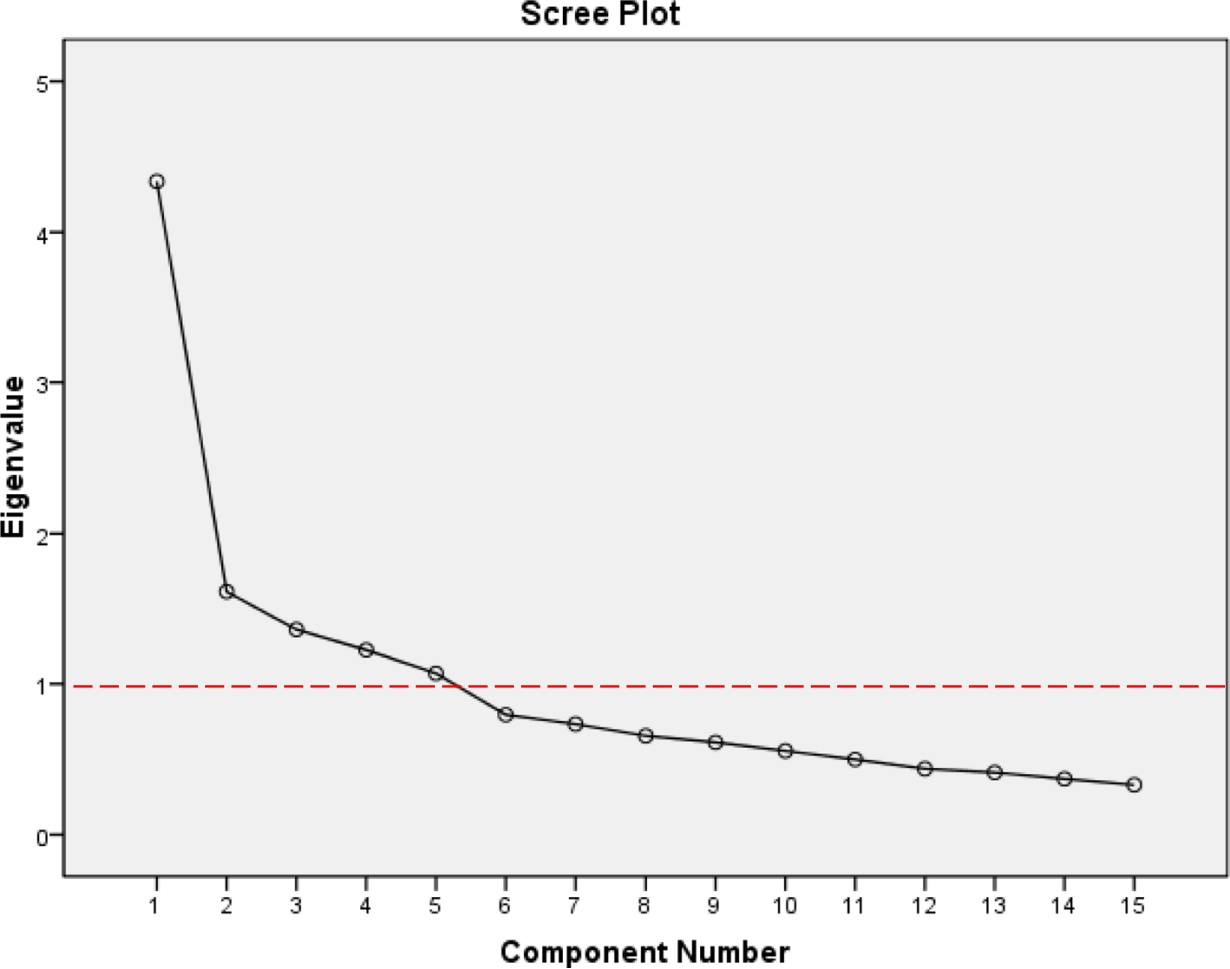
Scree plot exploratory factor analysis

Moreover, the Scree plot, as shown in Fig. 2, indicates that the highest factor loading is assigned to the first component, and a total of 5 components can be observed in this diagram (Fig. 2).

#### Varimax rotation

Based on the results, 5 extracted factors explained 64.07% of the variance of health media messengers. The amount of this variance and the alignment of the results with the theoretical foundations, previous research and the qualitative section indicate the appropriate validity of the obtained criteria. Before the factor analysis and based on the nature of the media messengers, experts' opinions and theoretical foundations, the items were classified into 6 categories as shown in Table 2. However, the factor analysis based on Table 7 clarified that there are two categories of Formal information sources of the Ministry of Health (official) and the Informal conversation (those obtained through talking with friends, colleagues and classmates, and talking in the family, friends and relatives) and should be placed in one category (Table 7).

**Table 7:** Rotated component matrix and factor loads of UHMQ questions

Items	Health Messengers in order of Importance (Priorities)				
	Mobile application and social networks	Medical universities formal/informal news	Academic resource	Internet search	Mass media
	1	2	3	4	5
Q10. Health-related groups/Channels on WhatsApp	0.71				
Q11. Health-related groups/Channels on Instagram	0.79				
Q12. Health-related groups/Channels on Telegram	0.85				
Q13. Health-related groups/Channels on local messengers	0.47				
Q3. Health messages and news from the Ministry of Health (SMS)		0.75			
Q4. The website of the SUMS or the Ministry of Health		0.63			
Q14. Conversation and exchanging news with the family		0.69			
Q15. Conversation and exchanging news with colleagues and classmates		0.60			
Q1. Scientific books and articles			0.78		
Q2. Scientific conferences, webinars and meetings			0.80		
Q8. Search health related websites				0.75	
Q9. Search for information on Google				0.82	
Q5. Radio					0.80
Q6. Television					0.67
Q7. Public Magazines and newspapers					0.59

## Discussion

**Fig. 3:**
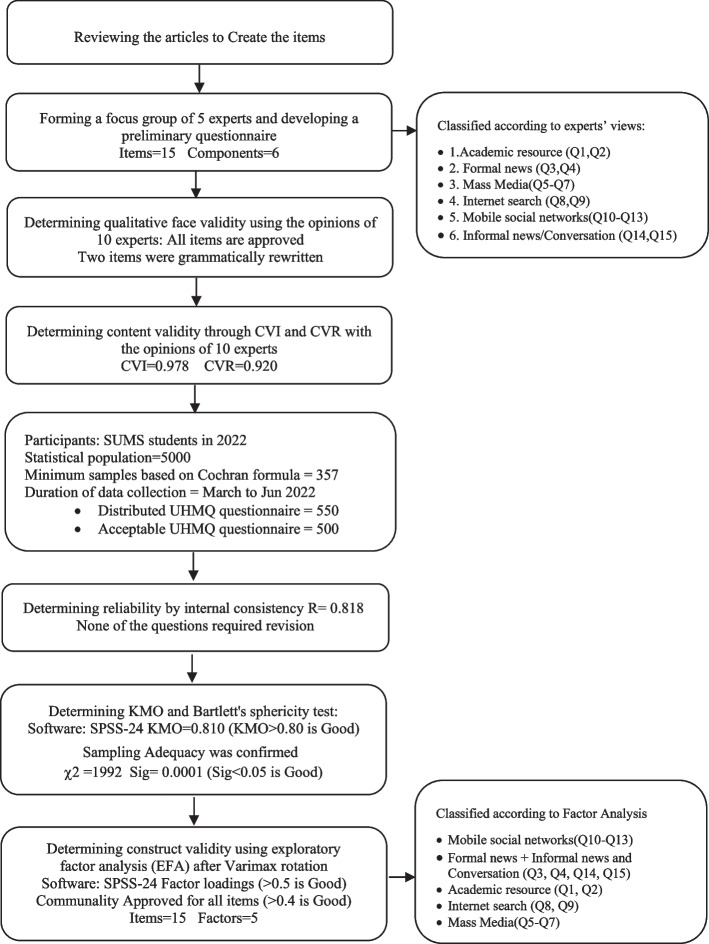
Research steps in developing the questionnaire (Use of Health Messenger Questionnaire)

In the qualitative stage of this research and based on the opinion of experts and theoretical bases, health media messengers were categorized into 6 components and 15 items, and then by using exploratory factor analysis, the validity and reliability of the instrument were examined. A summary of the results is illustrated in Fig. 3.

### Psychometric analysis of instrument components

In the initial analysis of items of the questionnaire, based on face content validity, which was conducted by a survey of experts, 15 items were confirmed and all the items in the qualitative section were also verified. In the content validity phase, the results showed that CVI was confirmed favorably with the indicators of simplicity (0.973), clarity (0.987) and relevance (0.973) and the cumulative average (0.978). In addition, item analysis revealed that all the items had a score of over 0.80, which manifested the content validity of the items. Inasmuch as the number of experts who participated in the confirmation of content validity was 10, based on the content validity indicator provided by Waltz and Bausell (1981), the validity of the whole questionnaire and each questionnaire item have been confirmed to a great extent [38]. In the CVR, the degree of necessity of each item was reviewed, and based on Lawshe's content validity ratio, when the opinions of 10 experts are collected, the average agreement of the opinions is expected to be at least 62% [31]. In the current research, the total average was 0.920, and more than 80% was confirmed for each item except for item number 13. The only item with a little agreement on its necessity was "Using local messengers and social networks" where in the agreement rate was 0.4. Of course, due to its confirmation using CVI, there was a necessity to compare it with other social networks, we kept the item in our study to examine it separately in the construct validity phase.

Regarding the reliability of the tool, we checked the internal consistency of the questions using Cronbach's alpha, and the internal consistency of the whole questionnaire was 0.818. Cronbach's alpha is one of the most common tests to measure the internal consistency of questions and to determine the reliability, especially in Likert scale questionnaires [44, 45]. The amount of Cronbach's alpha index ranges from 0 to 1, and the closer it is to 1, the greater internal consistency it has with the dimensions of the questionnaire [46–49].

The reliability of the whole questionnaire is expected to be at least 0.70 and the amount between 0.8 and 0.9 is excellent [50, 51]. Moreover, in the If Item Deleted mode of the SPSS software, each item was deleted one by one in order and the reliability of the rest of the items was calculated. If the reliability increases by removing an item, the item is problematic and we can edit or delete the option. As shown in the reliability results of the questionnaire, by removing each item, the reliability of the rest of the questions decreased which indicated that each items had a positive effect on the overall reliability. Further, when the reliabilities of sub-groups and sub-scales were calculated, the highest reliability was related to the subscale of Mobile-based social networks (0.761) and the lowest was connected with mass media (0.633). Another significant point in the analysis of Cronbach's alpha was that the value of Cronbach's alpha is not only affected by the internal consistency of the questions, but also by the number of questions, namely the less the number of questions, the lower the Cronbach's alpha [51, 52]. If the number of questions in the present questionnaire increases, it is expected that the reliability will also increase.

But the most important part of determining the validity of the instrument was calculating the construct validity using factor analysis. The validity of the instrument was confirmed based on the significant statistical indicators and the factor analysis. With KMO = 0.810 and Bartlett's sphericity < 0.001, the adequacy of the sample size and the appropriateness of the test were corroborated. Basically, the value of KMO > 0.80 is a good indicator [40–42].

Likewise, according to Table 5, communalities explained by each of the variables exceeded 0.6. The minimum expected value for this index is 0.5 [44] and 0.4 and 0.3 values are also acceptable in some articles [44]. So the value 0.6 is a suitable index. In accordance with the initial classification of the quantitative phase which was based on the essence of the items, 15 items in 6 categories of academic messengers (books and articles), official messengers (website and text messages of the Ministry of Health and Universities of Medical Sciences), internet search, Social media, common mobile-based social networks (WhatsApp, Telegram, Instagram and Iranian social networks) and informal methods (conversation with colleagues, classmates and family) were categorized. Severin & Tankard (2010) also put formal and informal methods in two separate categories [35], but in the analysis of the exploratory factor analysis of this research, the two formal and informal categories were merged together and placed in one category.

Although, at first glance, these two categories look dissociated in terms of title, the information of the government sources of the Ministry of Health (the website of the University of Medical Sciences and the notification messages of the Ministry of Health) is included in the Medical Sciences category. On the other hand, the category of informal sources in this research was defined by two items (talking with friends, colleagues, and classmates, and talking with the family, friends, and relatives). It should be noted that our population and statistical sample are from students of medical sciences. Accordingly, most of their friends, colleagues and classmates are also students of the University of Medical Sciences. Therefore, the combination of these two categories (Formal /Informal) can probably be delineated by the construct "The Context of the University of Medical Sciences". In fact, if our statistical population is not from the students of the aforementioned university, these two areas may be separated. Once again, we conducted the exploratory factor analysis with the assumption of 6 factors. Interestingly, these two areas were separated according to theoretical bases and finally 6 predicted categories were confirmed. Additionally, the present questionnaire covers more than 0.64 of the concept of health messengers. We checked the messaging methods that were more common, and some methods such as digital boards installed in the city are methods that were not mentioned in the study.

### Analysis of students’ preferences in selecting a messenger (factor priority)

The second part of the results deals with the analysis of students' preferences in media selection, which was based on the factor load of the components. On the whole, five factors were extracted in the factor analysis of the questionnaire which explained about 64% of the construct according to Table 6.

#### Social networks and messenger applications

Messengers based on social media was the first messenger factor that had the highest factor load and was the students’ first choice. Similar research has reported that medical students are busy either in the classroom or in clinical settings and have access to smartphones more than anything else. Therefore, it can be predicted that they mostly have access to the news of health messages through mobile phones rather than anything else [53–55].

Raiman et al. (2017) found that one of the reasons for the preference of social media among the students is the availability and convenience of using instant messages; the immediate benefits of messaging to enhance understanding and learning, and the ability to access recorded discussions and using “voice” to ask questions.

In fact, in addition to being available, it is a two-way communication with feedback [54]. The factors of age and generation are also effective. In his research, Kubrick showed that the preferences of old people are different when compared to young people regarding the choice of messengers [56]. Inasmuch as students mostly belong to the digital age, they prefer electronic devices and feel comfortable with them. Moreover, mobile-based approaches are their priorities and had the highest factor load.

Among the items of this component, four messenger of Instagram, WhatsApp, Telegram and Iran’s national messenger were examined. The highest factor load was related to Telegram (0.85), followed by Instagram (0.79), WhatsApp (0.71) and local messengers (0.47). If we deleted national messengers from the questionnaire, the impact of this factor would be greater. The reason why Telegram was preferred was probably due to the capabilities of the social network of Telegram in transferring different pictorial and textual contents and the recovery of files in Telegram. For example, there are some membership limits for channels and groups in WhatsApp, while Telegram enjoys a better status. Moreover, speed and technical problems of national social networks influences people’s choice. The preference of international over national networks may be because of the open virtual environment which is not limited to a particular time or space, is used by all and is not localized. It seems that mobile social media will not disappear in the near future. Therefore, we should ponder extensively over mobile infrastructure as well as how individuals incorporate them into their everyday lives [57].

#### Formal and informal messengers

The sources of health knowledge are divided into two categories; formal (official) sources (doctors and health service providers) and informal sources (family members) [1]. However, in the current research, the second factor in the health messaging questionnaire was based on the opinions of medical students, government messengers affiliated with the Ministry of Health, and informal messaging through colleagues, classmates, friends, and family.

Though at first look the two groups look far apart, as conversational information which is exchanged among friends and families of students of universities of medical sciences come from resources affiliated with those universities. Therefore, integration of the two factors can be labelled as ‘University of Medical Sciences setting’. It is crystal clear that in the aforementioned setting and the health affiliated fields the websites of the Health Ministry and universities of medical sciences are well-liked and placed as the main page on students’ personal computers as most of the university news is broadcast via them. On the other hand, university students follow many protocols, guidelines and news from the university site; thus, this component is among the highest priorities and the foregoing students share the news with their classmates, colleagues and families. It seems that in those studies the population of which come from universities other than universities of medical sciences nor from the related fields, the two areas are separate and independent. This point needs to be further checked in other studies. For example, in a research study it was illuminated that the first health messenger used was TV and Iran health networks were preferred followed by specialists, public newspapers, radio and internet. Satellite channels were on the bottom of the list. They also found that, although mass messengers may be the first source of news via which many people receive information, they do not suffice and people prefer face to face interpersonal communication to supplement or ensure the accuracy of information [3]. In other words, the combination of collective information (one-way) along with individual face to face communication is considered a more complete method, and people are eager to investigate practical experiences of others on the official news they hear.

#### Academic resources

One of the sources of receiving health information is academic sources such as experts, books, etc. [35]. In the current research, the third health messenger factor is academic resources such as academic conferences, books and articles. The rank of this component can be affected by the type of research sample. Inasmuch as books and academic resources are examined in the classrooms and some of the valid academic information is reviewed in the journals of educational clubs and seminars; therefore, access to university professors can all affect the selection and priority of academic resources. Wilson believes that a wide range of emotional and cognitive attractions are used in the field of health. Logical and informative messages emphasize knowledge and convince the audience by stating facts, forms and information (for example, facts related to AIDS, its causes, transmission routes and prevention methods). Educated people mostly search messages which not only augment awareness and knowledge but also have academic credit, while less educated ones are attracted by appealing messages [36].

#### Searching the internet

The fourth factor in receiving health information is searching on the internet and free resources in search engines. In the era of technology, searching on the internet is one of the most important ways of obtaining information among people. In terms of availability, the internet is one of the main tools in searching for information [58, 59]. Research has laid out that most people use the internet to search for health information [60–66]. In this research, searching on the internet was the fourth factor. This is due to the fact that medical students are exposed to more reliable sources such as academic sources and they have access to the website of the Ministry of Health whereby they receive reliable information However, smart phones increase the possibility of searching on the internet [65].

#### Mass media

According to the results of the research, the fifth messenger factor is mass media such as radio, television and publications. Today, due to the availability of electronic publications, social media and social channels related to health which are more accessible, the use of paper and printed methods has diminished. Since our sample was recruited from medical students who have greater access to more reliable sources such as professors, specialists, the and websites of the university and the Ministry of Health. In healthcare environments such as hospitals and clinics, access to televisions or radios may be limited for medical students and trainees. However, with the widespread availability of internet access and mobile devices, searching online has become a more accessible and convenient option for obtaining information and learning. As such, digital resources and online platforms have become increasingly important for medical education and training.

However, different groups have different preferences. For example, in Razavi et al.'s (2016) research, among health messengers, radio, television, individual face-to-face training, newspapers and magazines, CDs training, holding training courses in the form of pamphlets, were announced to be the highest priority [67]. It seems that "availability" in time and space is an important point in choosing media. For example, in the case of ordinary people like housewives, TV is the highest priority, but for a driver or an employer with an average education, radio is a suitable choice. Among people with specialized education, news is more important due to greater availability of specialists and reliable sources.

Interestingly, in our research, students used both official and unofficial news. In Khaniki's research, after receiving news from television and radio sources, individuals’ next priority was interpersonal communication [3] accounting for the fact that we need face to face communication as communication is an important act of interaction [68] and we have an interest in sharing with others what we hear (dissemination) or testing its validity. Exchanging messages may be a means to interpersonal communication.

## Conclusion

The findings of the present study clarified that medical students who have greater access to and deeper awareness of reliable sources, have wiser choices while selecting messengers. On the other hand, social media enjoys greater popularity in terms of transmitting messages which shows that students prefer messengers free from time and space restrictions; the ones which are constantly available. Therefore, to develop health knowledge and awareness, mobile-based methods or enrichment of social media are students’ top priorities. It seems that ease of access to common and international social media can be more effective in improving health knowledge in the society. Moreover, it is deemed necessary to enrich and periodically update medical science websites.

Medical students and healthcare professionals are often on the move due to the nature of their work. They often prioritize mobile-based methods and prefer messengers that are free from time and space restrictions. The widespread availability of mobile devices and the ability to search for and access health information make it easier to test health information via mobile devices. Therefore, in health policy, attention should be paid to virtual capabilities, especially mobile-based approaches. The questionnaire used in the present study can be a valid tool for measuring individuals' preferences in selecting messengers. However, due to the specialization of health knowledge and the vital need to acquire it, the results yielded by the present questionnaire are likely to be affected by participants' basic information. Future research can focus on investigating the effectiveness of mobile-based methods and social media in developing health knowledge and awareness among medical students and the general population.

### Strengths and limitations of the study

This questionnaire was conducted on 500 participants and the data are rich in terms of saturation. The research instrument is novel and its validity has been confirmed with appropriate indicators. Therefore, it can be used in further studies. This work has been limited to medical students who are part of the health workforces. The implementation of this research among other sectors of society may yield different results and individuals’ preferences may vary depending on specialized and non-specialized basic knowledge. Moreover, all participants were students of medical sciences, therefore, the effect of the diversity of the level of education was not feasible. We examined common messengers in transmitting health messages. It is suggested bulletins, digital boards, etc. be added to questionnaires in further studies.

## Data Availability

The datasets used and/or analyzed during the current study are available from the corresponding author on reasonable request.

## Abbreviations

SUMS: Shiraz University of Medical Sciences
UHMQ: Using Health Messengers Questionnaire
CVI: Content Validity Index
CVR: Content Validity Ratio
